# Co-Complexes-Based Self-Oscillating Gels Driven by the Belousov–Zhabotinsky Reaction

**DOI:** 10.3390/gels10090552

**Published:** 2024-08-26

**Authors:** Ilya L. Mallphanov, Michail Y. Eroshik, Dmitry A. Safonov, Anastasia I. Lavrova

**Affiliations:** 1 Center for Nonlinear Chemistry, Immanuel Kant Baltic Federal University, 14 A. Nevskogo Street, Kaliningrad 236016, Russia; misha.eroshik@yandex.ru (M.Y.E.); dasafonoff@gmail.com (D.A.S.); aurebours@googlemail.com (A.I.L.); 2 Saint-Petersburg State Research Institute of Phthisiopulmonology, 2-4 Ligovsky Avenue, Saint-Petersburg 191036, Russia; 3 Medical Institute, Saint-Petersburg University, Universitetskaya Embankment 7/9, Saint-Petersburg 199034, Russia

**Keywords:** cobalt complexes, polymers, self-oscillating gels, Belousov–Zhabotinsky reaction

## Abstract

We report the synthesis of novel cobalt complexes-based catalysts designed for the oscillatory Belousov–Zhabotinsky (BZ) reaction. For the first time, we introduce cobalt complex-based self-oscillating gels that demonstrate autonomous color oscillations within a BZ reagent solution, functioning without the need for any external stimuli. We created acrylamide-based self-oscillating gels containing immobilized tris(2,2′-bipyridine)cobalt(II) or tris(1,10-phenanthroline)cobalt(II) complexes and gels containing covalently bound (5-acrylamido-1,10-phenanthroline)bis(2,2′-bipyridine)cobalt(II), (5-acrylamido-1,10-phenanthroline)bis(1,10-phenanthroline) cobalt(II), or tris(5-acrylamido-1,10-phenanthroline)cobalt(II) complexes. When the BZ reaction takes place within the gels, it results in the observation of moving chemical waves and reversible color changes. We believe that Co-complexes-based self-oscillating gels have potential applications in the design of soft actuators and chemical devices for signal processing.

## 1. Introduction

The synthesis and study of autonomous self-oscillating polymer gels, which periodically change their properties when the Belousov–Zhabotinsky (BZ) oscillatory reaction occurs in them, is an intensively developing scientific direction [1,2,3]. These materials have wide application prospects in such areas as the creation of autonomous soft actuators [1,3], micropumps [4], systems [5] for mass transport, and devices for processing information using chemical waves [6,7,8,9].

To synthesize self-oscillating gels, scientists incorporate ruthenium complexes, usually derivatives of tris(bipyridine)ruthenium(II) [Ru(bpy)_3_^2+^] that can catalyze the BZ reaction, into the polymer matrixes of the cross-linked polyacrylamide [10] or poly(*N*-isopropylacrylamide) gels [11,12,13]. The BZ reaction has a complex mechanism consisting of several dozen reactions, but in general, it can be represented as the bromination and oxidation of an organic substance [for example, malonic acid (MA)] with bromate in the presence of a catalyst [for example, Ru(bpy)_3_^2+^] in an acidic aqueous solution [14,15,16]. During the reaction, the catalyst periodically changes its oxidation state. If the catalyst is covalently bound to the polymer matrix of the gel, then the changes in oxidation state of the catalyst result in periodic changes in the properties (color, size, transparency) of the gel.

If such a poly(*N*-isopropylacrylamide) gel with immobilized catalytically active complex is immersed in an aqueous solution [catalyst-free BZ solution (CFBZ)] of all the BZ reagents except the catalyst, then the BZ reaction starts in the gel and provides periodic redox transitions of the complex. The change in oxidation state of the catalyst changes the volume phase transition temperature of the gel, as well as the degree of swelling, since the hydrophilicity of the gel increases when the catalyst is oxidized and decreases when it is reduced [17]. The gel swells/shrinks when the catalyst is in an oxidized/reduced state; these changes in the gel volume (chemomechanical oscillations) coincide with the redox oscillations of the complex.

Despite the wide popularity of the Ru complexes among researchers, their practical application for the synthesis of self-oscillating gels is limited since these complexes are expensive, difficult to synthesize, and photosensitive. Thus, at present, the search for new, inexpensive, reliable, photoinsensitive, and easily synthesized self-oscillating gels remains an urgent task.

Attempts have been made to synthesize self-oscillating gels based on complexes of iron (Fe complexes) [18,19,20,21,22]. However, the number of such gels is very small compared to those based on Ru complexes. To date, only gels based on Ru and Fe complexes have been reported. There is no mention in the scientific literature of gels based on other catalysts, such as Co complexes, for the BZ reaction, despite evidence that complexes of cobalt with 2,2′-bipyridine and 1,10-phenanthroline can also catalyze [23] this reaction.

Co complexes are cheap, easy to synthesize, photoinsensitive, and catalyze the BZ reaction under mild conditions, making them promising for creating self-oscillating gels.

In our work, we developed, for the first time, self-oscillating gels that generate and propagate chemical waves driven by the redox oscillations of Co complexes.

We synthesized the following complexes: tris(2,2′-bipyridine)cobalt(II) sulfate [Co(bpy)_3_^2+^], tris(1,10-phenanthroline)cobalt(II) sulfate [Co(phen)_3_^2+^], (5-acrylamido-1,10-phenan-throline)bis(2,2′-bipyridine)cobalt(II) sulfate [Co(acphen)(bpy)_2_^2+^], (5-acrylamido-1,10-phenanthroline)bis(1,10-phenanthroline) cobalt(II) sulfate [Co(acphen)(phen)_2_^2+^], and tris(5-acrylamido-1,10-phenanthroline)cobalt(II) sulfate [Co(acphen)_3_^2+^].

The complexes were characterized by spectral methods; the compositions of the complexes were confirmed using elemental analysis.

Based on the obtained complexes, we synthesized two types of gels. 

The first type of gel was produced by immobilizing Co(bpy)_3_^2+^ or Co(phen)_3_^2+^ complexes within polyacrylamide-based cation-exchange resins. The second type was created through the copolymerization of Co(acphen)(phen)_2_^2+^ with acrylamide and *N*,*N*′-methylenebisacrylamide, or by copolymerizing Co(acphen)_3_^2+^ with acrylamide.

When subjected to the Belousov–Zhabotinsky (BZ) reaction, these gels exhibit distinct self-oscillatory behavior, observable through color microscopy. Digital analysis of the recorded data further confirms the presence of autonomous, periodic oscillations within the gels.

## 2. Results and Discussion

**Scheme 1:**
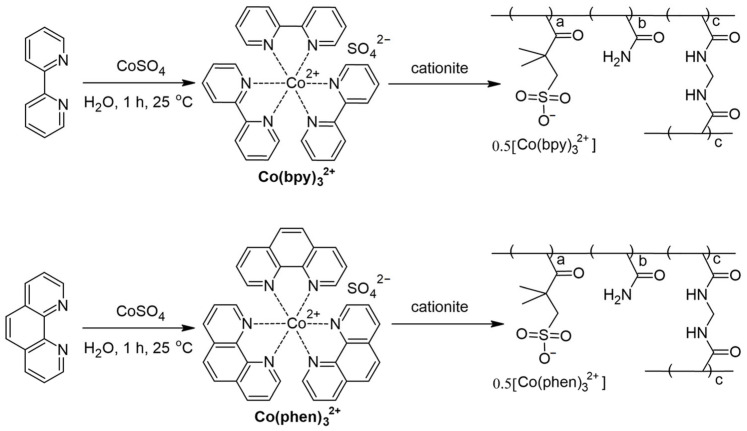
Synthesis of the complexes Co(bpy)_3_^2+^ and Co(phen)_3_^2+^ and their immobilization in the cationite.

First, the Co(bpy)_3_^2+^ and Co(phen)_3_^2+^ complexes (Scheme 1) were synthesized as reported in detail in Section 4.2 and Section 4.3. All the complexes synthesized in our work were obtained in the form of sulfates. The oxidation state of cobalt in all the synthesized complexes was 2+. The structure of Co(bpy)_3_^2+^ and Co(phen)_3_^2+^ complexes is well known [24,25]. Cobalt salts react with 2,2′-bipyridine and/or 1,10-phenanthroline to form complexes that have Co-ligand coordinate covalent bonds. In such complexes, each bidentate ligand donates its two lone pairs of electrons to the cobalt(II) ion. The both complexes have slightly distorted octahedron geometry.

Since the self-oscillatory behavior of gels is observed visually, primarily by periodic changes in the color (oxidation state) of the Co-based catalyst, we measured the spectra of Co(bpy)_3_ and Co(phen)_3_ before and after change (via an oxidizing agent) in cobalt’s oxidation state (from +2 to +3) in the complexes to understand whether these transitions can be observed in the visible region of the spectrum. To obtain the 3+ oxidation state of cobalt, we added NaBrO_3_ and H_2_SO_4_ to solutions of Co(bpy)_3_^2+^ and Co(phen)_3_^2+^.

Specifically, upon addition of 0.1 M NaBrO_3_ and 0.5 M H_2_SO_4_ (concentrations commonly used in BZ mixtures), a 1 mM solution of Co(bpy_)3_^2+^ changes from brownish-yellow to colorless (formation of Co(bpy)_3_^3+^), and a 1 mM solution of Co(phen)_3_^2+^ changes to a shade of yellow (formation of Co(phen)_3_^3+^). In dry form, Co(bpy)_3_^2+^ and Co(phen)_3_^2+^ are brown powders.

**Figure 1:**
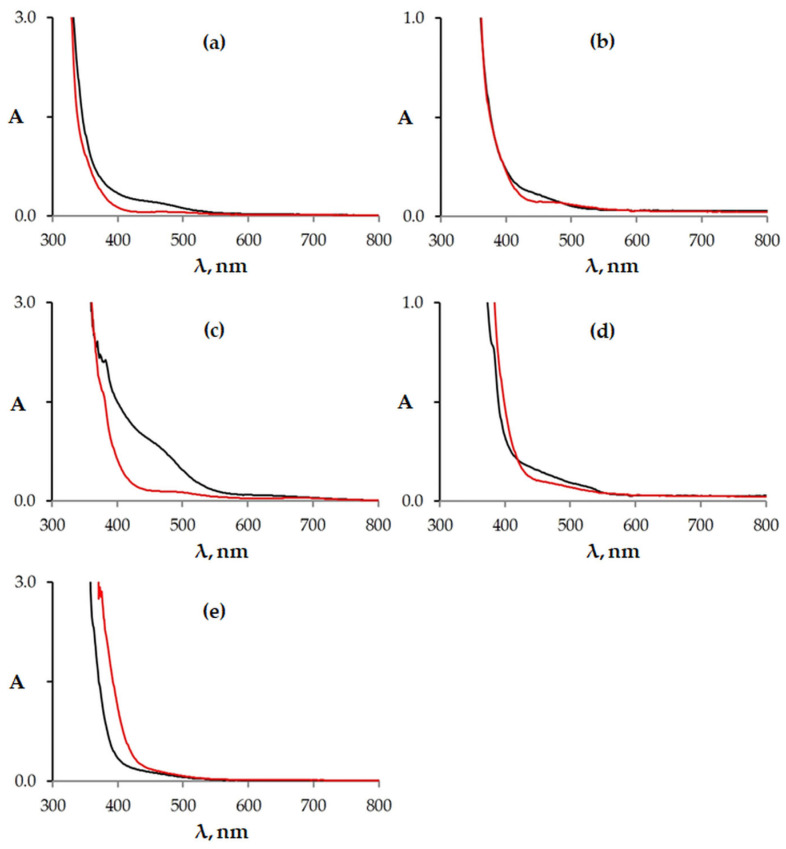
VIS spectra of (**a**) Co(bpy)_3_, (**b**) Co(phen)_3_, (**c**) Co(acphen)(bpy)_2_, (**d**) Co(acphen)(phen)_2_, and (**e**) Co(acphen)_3_ in reduced (Co^2+^) (black line) and oxidized (Co^3+^) (red line) states. Aqueous solutions of complexes: C = 1 mМ.

The spectra of Co(bpy)_3_^2+^ and Co(bpy)_3_^3+^ are shown in Figure 1a, and the spectra of Co(phen)_3_^2+^ and Co(phen)_3_^3+^ in Figure 1b. As can be seen in the visible region (400–800 nm) of the spectrum, for Co(bpy)_3_, the difference between the absorptions of the reduced and oxidized forms is small; for Co(phen)_3_, the spectra differ even less. The solutions of Co(bpy)_3_^2+^ and Co(phen)_3_^2+^ have no definite absorption bands in the visible region, but show inflections at about 450 nm. The comparison of Co(phen)_3_^2+^ and Co(phen)_3_^3+^ shows that the Co(phen)_3_^2+^ complex has no band in the visible region, while the Co(phen)_3_^3+^ complex has a smaller one at 466 nm. The same relation was observed in the case of Co(bpy)_3_ complexes; Co(bpy)_3_^3+^ has a band at 471 nm, while Co(bpy)_3_^2+^ has only an inflection of absorption curve at near the same position. Nevertheless, the redox transitions of both complexes are distinctly observable at sufficiently high concentrations and/or with an adequately long optical path length and can be recorded using a sensitive color camera. 

As is known from work [26] devoted to the study of cobalt complexes, electronic transitions of complexes are mainly derived from metal-to-ligand charge transfer (MLCT) for Co(bpy)_3_^2+^ and ligand-to-metal charge transfer (LMCT) for Co(bpy)_3_^3+^. We suppose that the difference in the spectra of the reduced (Co^2+^) and oxidized (Co^3+^) forms of the cobalt complexes Co(bpy)_3_ and Co(phen)_3_ may be due to a change in the nature of this transition. 

**Figure 2:**
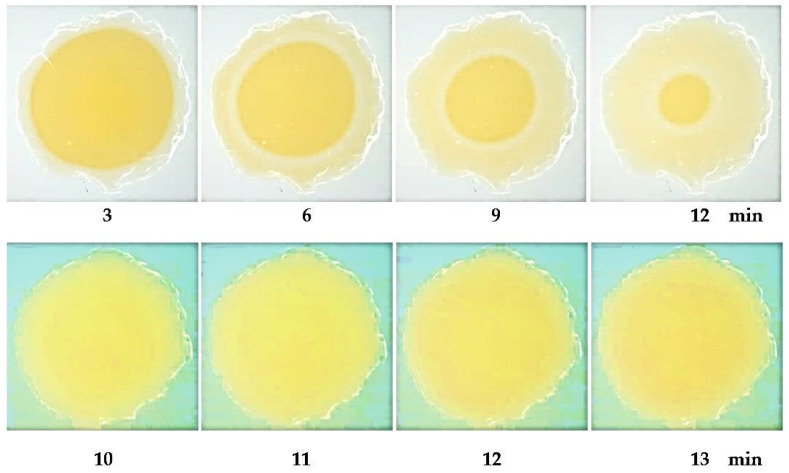
Snapshots showing the propagation of the BZ wave in the film of gel 1 (top). Snapshots of gel 2 color changes (bottom). CFBZ solution, concentrations of reagents M: MA = 0.05; NaBrO_3_ = 0.1; H_2_SO_4_ = 0.5.

To test Co(bpy)_3_^2+^ and Co(phen)_3_^2+^ as the catalysts for the oscillatory BZ reaction, each complex was immobilized in a film of a gel cationite (Scheme 1). The gel was synthesized by copolymerization of 2-acrylamido-2-methyl-1-propanesulfonic acid sodium salt with acrylamide using *N*,*N*′-methylenebisacrylamide as a cross-linking agent. For the procedures for the synthesis of the gel cationite and immobilization of the catalysts on it, see Section 4.7 and Section 4.8. As a result, the gel films (about 1 mm thick) containing immobilized catalysts were obtained. Figure 2 (top row, 3 min) shows a photo of the gel film (gel 1) with immobilized Co(bpy)_3_^2+^, and Figure 2 (bottom row, 10 min) shows a photo of the gel film (gel 2) with immobilized Co(phen)_3_^2+^. The concentrations of the catalyst in the films were about 50–70 mmol/L. 

To test the behavior of gels 1 and 2, the films were soaked in a CFBZ solution containing the following components: [NaBrO_3_] = 0.1 M; [MA] = 0.05 M; [H_2_SO_4_] = 0.5 M. The concentrations of reagents were selected experimentally.

These gels exhibit self-oscillatory behavior: chemical waves arise and propagate in gel 1 [Figure 2 (top row) and Movie S1]; gel 2 periodically changes color [Figure 2 (bottom row)]. This confirms the activity of the Co(bpy)_3_^2+^ and Co(phen)_3_^2+^ complexes as catalysts for the BZ reaction. Experimental setup used for recording the snapshots is given is Section 4.12.

In our case, the BZ reaction proceeds as bromination and oxidation of malonic acid with bromate in the presence of sulfuric acid and the Co complexes as the catalysts:

2BrO_3_^−^ + 3CH_2_(COOH)_2_ + 2H^+^ → 2BrCH(COOH)_2_ + 3CO_2_ + 4H_2_O

Taking into account the decomposition of bromomalonic acid, the balance of the BZ reaction can be written differently:

4BrO_3_^−^ + 3CH_2_(COOH)_2_ → 4Br^−^ + 9CO_2_ + 6H_2_O

We assume that in the case of using the Co complexes, the catalytic cycle is realized as in the case of using Fe complexes [27]:

BrO_3_^−^ + Br^−^ + 2H^+^ ⇄ HBrO_2_ + HOBr

HBrO_2_ + Br^−^+ H^+^ ⇄ 2HOBr

BrO_3_^−^ + HBrO_2_ ⇄ 2BrO_2_^•^ + H_2_O

BrO_2_^•^ + Co(L_n_)^2+^ + H^+^ ⇄ HBrO_2_ + Co(L_n_)^3+^

2HBrO_2_ ⇄ BrO_3_^−^ + HOBr + H^+^

Co(L_n_)^2+^ + BrMA → Br-^−^+ Co(L_n_)^3+^ + stable products.

However, due to the limited data [23] available in the scientific literature on the use of cobalt complexes to catalyze the BZ reaction, it is not yet possible to make a definitive conclusion regarding the mechanism of the BZ reaction involving these complexes.

The self-oscillating gels obtained by immobilizing catalytically active cations on the cationite have a significant drawback—the gels lose the catalysts when immersed in the CFBZ mixture with low pH values. Due to the leaching of the catalyst into the environment, the amplitude of the chemical waves in the gels gradually decreases, and after 20–30 min, they completely disappear. The transparent cationites are only suitable for testing new BZ catalysts and not for creating self-oscillating gels that must exhibit long-term oscillations.

**Scheme 2:**
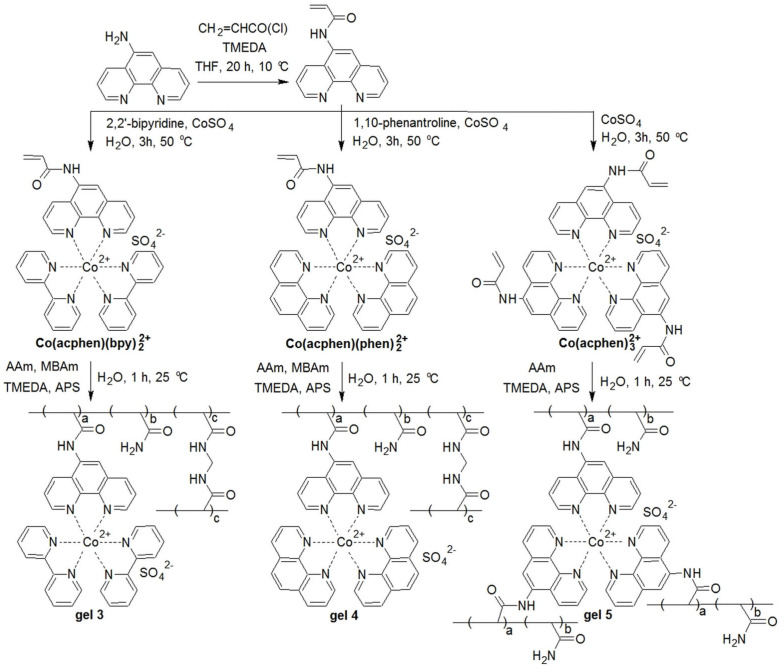
Synthesis of the complexes Co(acphen)(bpy)_2_^2+^, Co(acphen)(phen)_2_^2+^, and Co(acphen)_3_^2+^; gels 3, 4, and 5. Designations: TMEDA—tetramethylethylenediamine; THF—tetrahydrofuran; AAm—acrylamide; MBAAm—*N*,*N*′-methylenebisacrylamide; APS—ammonium persulfate.

Having proved that the Co(bpy)_3_^2+^ exhibits catalytic activity in the BZ reaction and shows relatively bright and easily visible color changes, we synthesized its derivative, heteroleptic complex Co(acphen)(bpy)_2_^2+^, containing two 2,2′-bipyridine ligands and one 1,10-phenanthroline ligand with acrylamide fragment (Scheme 2).

The synthesis is described in the Section 4.4. This acrylamide moiety was introduced into the complex to provide a possibility of copolymerization of this derivative with acrylamide and *N*,*N*′-methylenebisacrylamide. Such copolymerization may lead to the formation of the gels with covalently bound catalytic fragments. We chose the derivative of Co(phen)(bpy)_2_^2+^ rather than that of Co(bpy)_3_^2+^ for covalent binding to the polymer matrix since Co(phen)(bpy)_2_^2+^ is more stable in the strongly acidic environment of the BZ reaction than Co(bpy)_3_^2+^. In dry form, Co(phen)(bpy)_2_^2+^ is a brown powder. Upon the addition of 0.1 M NaBrO_3_ and 0.5 M H_2_SO_4_, a 1 mM solution of Co(acphen)(bpy)_2_^2+^ changes from brownish-yellow to yellow. The spectra of the complex Co(acphen)(bpy)_2_ in reduced (II) and oxidized (III) forms are shown in Figure 1c. 

**Figure 3:**
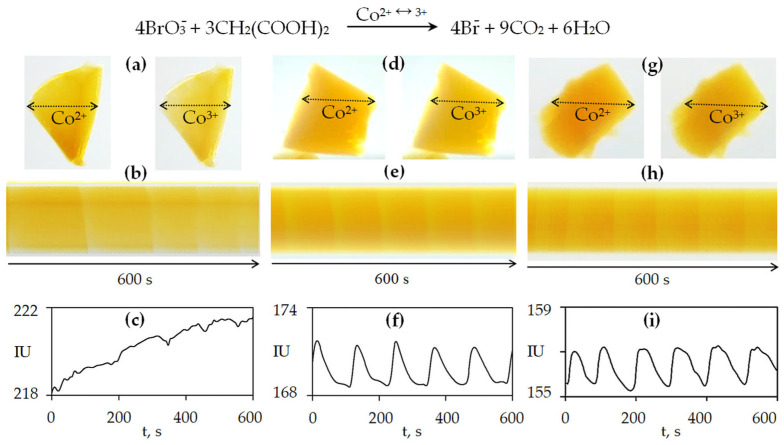
Oscillations of pieces of gels 3, 4, and 5 in the CFBZ solution. (**a**,**d**,**g**) Snapshots of pieces of gels 3, 4, and 5, respectively, in the reduced (Co^2+^) (left snapshot) and in the oxidized (Co^3+^) (right snapshot) states of the catalyst. (**b**,**e**,**h**) Space–time plots of pieces of gels 3, 4, and 5, respectively. For gels 3, 4, and 5, space–time plots are built along the arrows. In the space–time plots, vertical yellow/orange stripes present the oxidized(Co^2+^)/reduced(Co^2+^) state of the catalyst. (**c**,**f**,**i**) Dependence of IU (intensity unit) of the piece on time (s) for gels 3, 4, and 5, correspondingly. CFBZ solution, concentrations of the reagents M: MA = 0.05; NaBrO_3_ = 0.1; H_2_SO_4_ = 0.5.

As can be seen, the difference between the absorptions for Co(acphen)(bpy)_2_^2+^ and Co(acphen)(bpy)_2_^3+^ is significantly greater than those for Co(bpy)_3_ or Co(phen)_3_. We think that this difference in the spectra between Co(acphen)(bpy)_2_^2+^ and Co(acphen)(bpy)_2_^3+^ is due to the destruction of the complex during its oxidation in the acidic environment. By copolymerizing the Co(acphen)(bpy)_2_^2+^ complex with acrylamide and *N*,*N*′-methylenebisacrylamide, we synthesized gel 3 (Scheme 2), as depicted in Figure 3a (details of the synthesis are provided in Section 4.9.

In gel 3, the Co(acphen)(bpy)_2_^2+^ moiety acts as a catalyst, acrylamide forms the polymer chains, and *N*,*N*′-methylenebisacrylamide cross-links them.

Gel 3 was synthesized in the form of a cylinder (1 × 1 cm^2^) containing about 2.7 mole% of Co(acphen)(bpy)_2_^2+^ relative to acrylamide. After soaking gel 3 in distilled water to remove residual unreacted monomers, it was obtained as a yellow-brown material that did not discolor in either water or acid solution (H_2_SO_4_ = 0.5 M), indicating covalent bonding of the catalyst to the polymer matrix. The obtained cylinder was divided into separate (submillimeter) pieces for studying of the oscillatory and chemomechanical properties.

To test the oscillatory behavior of gel 3, a piece of the gel (Figure 3a) was immersed in the aqueous CFBZ solution (see details in Section 4.12). The concentrations of bromate, malonic acid, and sulfuric acid were the same as for gels 1 and 2.

When the BZ reaction occurs, the piece of gel 3 shows weak color (Figure 3b and Movie S2) oscillations that decay with time; chemical waves arise in the gel and propagate throughout the piece. Its self-oscillatory behavior is presented in the space–time plot (Figure 3b) and IU-t graph (Figure 3c). The space–time plot shows the dependence of the gel piece’s color on time. The experimental setup to observe and record the snapshots of the gels as well as procedures for building the space–time plots and IU-t graphs are given in Section 4.11. 

The gel becomes lighter when the Co(acphen)(bpy)_2_ moiety is in an oxidized state (Co^3+^) and darker when the complex is in a reduced state (Co^2+^). Although gel 3 exhibits oscillations, their amplitude is small, and the oscillations rapidly diminish. We attribute this to the gradual degradation of the heteroleptic 2,2′-bipyridine-containing complex Co(acphen)(bpy)_2_^3+^ in the oxidized and acidic environment.

To increase the stability of the complex in the BZ reaction, we synthesized a Co(acphen)(phen)_2_^2+^ complex consisting of three 1,10-phenanthroline ligands, one of which contains acrylamide fragment (see Scheme 2). In dry form, Co(acphen)(phen)_2_^2+^ is a brown powder. Upon the addition of 0.1 M NaBrO_3_ and 0.5 M H_2_SO_4_, a 1 mM solution of Co(acphen)(phen)_2_^2+^ changes to a shade of yellow (formation of Co(acphen)(phen)_2_^3+^). The spectra of the solutions of the complex Co(acphen)(phen)_2_ in reduced(Co^2+^) and oxidized(Co^3+^) forms are shown in Figure 1d. As can be seen, the absorption difference between Co(acphen)(phen)_2_^2+^ and Co(acphen)(phen)_2_^3+^ is slightly greater compared to that for Co(phen)_3_. The solutions of Co(acphen)(phen)_2_^2+^ and Co(acphen)(phen)_2_^3+^ have no definite absorption bands in the visible region. We think that the difference in the spectra of the reduced and oxidized forms of Co(acphen)(phen)_2_ may be due to a change from an MLCT for Co(acphen)(phen)_2_^2+^ to LMCT for Co(acphen)(phen)_2_^3+^.

Copolymerizing the Co(acphen)(phen)_2_^2+^ complex with acrylamide and *N*,*N*′-methylenebisacrylamide, we synthesized gel 4 (Scheme 2), shown in Figure 3d (see details of this syntheses in Section 4.10). In gel 4, the Co(acphen)(phen)_2_^2+^ moiety acts as a catalyst, acrylamide forms the polymer chains, and *N*,*N*′-methylenebisacrylamide cross-links them. 

Gel 4 was obtained in the form of a cylinder (1 × 1 cm^2^) and cut into approximately millimeter-sized pieces. After soaking the gel in distilled water to remove any residual unreacted monomers, it was obtained as a yellow material. The gel pieces did not lose color when soaked in water or acid solution (H_2_SO_4_ = 0.5 M), which may serve as confirmation of the covalent binding of the complex to the polymer network. Gel 4 contains about 2 mole% of Co(acphen)(phen)_2_^2+^ relative to acrylamide.

For testing gel 4, a piece of the gel was immersed in CFBZ solution (see details in Section 4.12). The concentrations of bromate, malonic acid, and sulfuric acid were the same as for gel 3.

When the BZ reaction occurs, the piece of gel 4 demonstrates self-oscillatory behavior (see space–time plot in Figure 3e). The piece turns yellow when Co(acphen)(phen)_2_ is oxidized (Co^3+^) and orangish when it is reduced (Co^2+^). Gel 4 demonstrates stable oscillations, the color of the piece changes periodically (Figure 3f) for a long time without significant weakening of oscillations. 

Inspired by the work of muscles, which convert chemical energy into mechanical movement, researchers are trying to create autonomous self-oscillating polymer gels that can mimic the functioning of muscle tissue. It is known that the cytoskeleton of muscle cells has myosin motors as the active cross-linkers to cross-link actin filaments [28]. Scientists applied the principles of cytoskeleton structure to synthetic gels and synthesized gels cross-linked with Ru complexes that catalyze the BZ reaction; the resulting gels demonstrated chemomechanical oscillations under BZ reaction conditions [29,30].

To implement this approach in the case of our Co complex-based gels, we synthesized Co(acphen)_3_^2+^ (Scheme 2), which can play the role of both catalyst and cross-linker. The complex was synthesized as reported in detail in Section 4.6. In dry form, Co(acphen)_3_^2+^ is a brown powder. Upon the addition of 0.1 M NaBrO_3_ and 0.5 M H_2_SO_4_, a 1 mM solution of Co(acphen)_3_^2+^ changes to a shade of yellow (formation of Co(acphen)_3_^3+^). The spectra of the solutions of the complex Co(acphen)_3_ in reduced (II) and oxidized (III) forms are shown in Figure 1e. Even though the solutions of Co(acphen)_3_^2+^ and Co(acphen)_3_^3+^ have no definite absorption bands in the visible region, the redox transitions of Co(acphen)_3_ are visible and can be detected. We think that the difference in the spectra of the reduced and oxidized forms of Co(acphen)_3_ may be due to a change from an MLCT for Co(acphen)_3_^2+^ to LMCT for Co(acphen)_3_^3+^.

Copolymerizing the complex Co(acphen)_3_^2+^ with acrylamide, we synthesized gel 5 (Scheme 2) (details of the synthesis in Section 4.11). In gel 5, complex Co(acphen)_3_^2+^ is used as a catalytic and cross-linking moiety, and acrylamide is used as a monomer that forms polymer chains. 

The chemical structure of gel 5 is shown in Scheme 2, while a snapshot of a piece of this gel is exhibited in Figure 3g. Gel 5 contains about 1.5 mole% of Co(acphen)_3_^2+^ relative to acrylamide. Gel 5 was obtained as a cylinder (1 × 1 cm^2^) and cut into approximately millimeter-sized pieces. After washing the gel of unreacted monomers, it was obtained as a yellow material that did not discolor in either water or acid solution (H_2_SO_4_ = 0.5 M), indicating covalent bonding of the catalyst to the polymer matrix.

The piece of gel 5 exhibits oscillations in the CFBZ solution (Figure 3h), becoming yellow [orange] in the oxidized (Co^3+^) [reduced (Co^2+^)] state of the catalyst. As can be seen from the graph (Figure 3i), the piece of gel 5 demonstrates stable long lasting oscillations. Also, the piece of gel 3 shrinks (swells) slightly when the catalyst is in an oxidized (reduced) state. The periodical changes in the linear size of the piece of gel 3 are very small (less than 0.5%) and difficult to detect. 

It is known that polyacrylamide-based self-oscillating gels, containing covalently bound or immobilized Ru- or Fe-based BZ catalysts, also change color and shrink/swell when the catalyst is in an oxidized/reduced state [10,20,31].

Researchers studying polyacrylamide-based self-oscillating gels have proposed that this behavior arises from the increased charge in the oxidized catalyst, which leads to stronger interactions with the polar side chains of the gel. During oxidation, additional reversible physical cross-links are formed, which are broken upon reduction. The formation of additional cross-links during oxidation of the catalyst generates osmotic pressure, pushing water out of the gel matrix, resulting in shrinkage of the gel. 

We believe that a similar mechanism occurs in acrylamide-based gels containing covalently bound Co-based catalysts: when the Co complex is oxidized (Co^3+^), additional cross-links form, leading to gel contraction. Upon reduction (Co^2+^), the cross-links are eliminated, and the gel returns to its original size. This phenomenon requires further, more detailed study.

Thus, from the obtained gels 1–5, only gels 4 and 5 showed stable self-oscillating behavior, demonstrating long-term oscillations. Gels 1, 2, and 3 lost the catalysts in the CFBZ solution and their oscillations quickly disappeared with time. Due to this, it is not possible to compare the catalytic activity of the complexes Co(bpy)_3_^2+^, Co(phen)_3_^2+^, and Co(acphen)(bpy)_2_^2+^ in gels 1–3 or outline possible differences in the mechanism of the BZ reaction based on the structure of the ligands.

Comparing the catalytic activity of the complexes Co(acphen)(phen)_2_^2+^ and Co(acphen)_3_^2+^ based on the oscillation periods of gels 4 and 5, respectively, we can conclude that the obtained catalysts for the BZ reaction under the same conditions exhibit approximately the same activity. For both the gels, the oscillation periods are about 100–120 s, which indirectly indicates the same catalysis mechanism.

## 3. Conclusions

We created, for the first time, Co complex-based self-oscillating gels demonstrating oscillatory behavior under the conditions of the Belousov–Zhabotinsky (BZ) reaction. The self-oscillating gels were synthesized by either immobilizing tris(2,2′-bipyridine)cobalt(II) or tris(1,10-phenanthroline)cobalt(II) in cationite, or by copolymerizing (5-acrylamido-1,10-phenanthroline)bis(1,10-phenanthroline)cobalt(II) or (5-acrylamido-1,10-phenanthroline)bis(2,2′-bipyridine)cobalt(II) with acrylamide and *N*,*N*′-methylenebisacrylamide. These gels, containing Co complexes covalently bound to a cross-linked polyacrylamide matrix, periodically change color during catalyst redox transitions. Additionally, we developed a tris(5-acrylamido-1,10-phenanthroline)cobalt(II) cross-linked acrylamide-based self-oscillating gel, in which the complex serves as both the catalyst and the cross-linker. This Co complex cross-linked gel exhibits stable and long-lasting self-oscillatory behavior. These gels can be used for development of actuators and chemical devices for signal processing.

## 4. Materials and Methods

### 4.1. Materials

For the synthesis, the following (analytical-grade) chemicals from Aldrich were used: 2,2′-bipyridine, acryloyl chloride, 2,2′-bipyridine, 1,10-phenanthroline, 1,10-phenanthroline-5-amine, acrylamide, *N*,*N*′-methylenbisacrylamide, cobalt(II) sulfate, tetrahydrofuran, benzene, ethanol, 2-acrylamido-2-methyl-1-propanesulfonic acid sodium salt, tetramethylethylenediamine, ammonium persulfate, malonic acid, sodium bromate, sulfuric acid, and distilled water. Chromatographic purification of substances was carried out using silica gel 60 (Aldrich) and Sephadex LH-20 (GE HealthCare Technologies, Inc., Chicago, IL, United States) as the sorbents. Proton nuclear magnetic resonance spectra of the obtained substances were recorded on a Bruker Avance III 400 MHz at temperature 23 °C in the Fourier transform mode; the chemical shifts were reported in ppm downfield from tetramethylsilane. To describe ^1^H NMR spectra, the following abbreviations were used: δ = chemical shift expressed in parts per million (ppm) by frequency; J = spin–spin coupling constant stated in Hz; s = singlet; d = doublet; t = triplet; m = multiplet or multiple signals; dd = double doublet. (CD_3_)_2_SO and D_2_O were used as a solvent for the ^1^H NMR studies. Visible absorption spectra were measured on a UV-3100 spectrophotometer (Shanghai Mapada Instruments Co., Ltd., Shanghai, China). Elemental analysis was conducted using microanalysis methods.

### 4.2. Synthesis of Tris(2,2′-bipyridine)Cobalt(II) Sulfate Heptahydrate [Co(Bpy)_3_^2+^]

For this synthesis, 2,2′-bipyridine (156.2 mg = 1 mmol) was dissolved in 10 mL of methanol. The resulting solution was mixed with 333 μL of 1 M aqueous solution of cobalt(II) sulfate (51.67 mg = 0.333 mmol) while stirring. The obtained mixture was kept stirring for 3 h at 50 °C and evaporated in vacuum. The obtained brown residue was dissolved in H_2_O and purified from unreacted compounds using column chromatography (Sephadex LH-20, H_2_O). Water solution was evaporated to give Co(bpy)_3_^2+^ (172.3 mg = 0.276 mmol) as a brown powder with an 83% yield. 

The parameters of the ^1^H NMR spectra: 8.69 (d, J = 8.0 Hz, 6H, bpy), 8.4 (t, J = 8 Hz, 6H, bpy), 7.64 (t, J = 6.8 Hz, 6H, bpy), 7.32 (d, 1H, J = 5.6 Hz, 6H, bpy).

Elemental analysis data for C_30_H_24_N_6_SO_4_Co × 7H_2_O (%): calculated C 48.06, H 5.11, N 11.21; found C 47.3, H 4.96, N 11.00. 

The VIS spectra of Co(bpy)_3_^2+^ and Co(bpy)_3_^3+^ are exhibited in Figure 1a.

### 4.3. Synthesis of Tris(1,10-phenanthroline)Cobalt(II) Sulfate Heptahydrate [Co(phen)_3_^2+^]

For this synthesis, 1,10-phenanthroline (182.2 mg = 1 mmol) was dissolved in 10 mL of methanol. The resulting solution was mixed with 333 μL of 1 M aqueous solution of cobalt(II) sulfate (51.67 mg = 0.333 mmol) while stirring. The obtained mixture was kept stirring for 3 h at 50 °C and evaporated in vacuum. The obtained brown residue was dissolved in H_2_O and purified from unreacted compounds using column chromatography (Sephadex LH-20, H_2_O). Water solution was evaporated to give Co(phen)_3_^2+^ (85.0 mg = 0.8 mmol) as a brown powder with an 80% yield. 

The parameters of the ^1^H NMR spectra: 8.94 (d, J = 8.4 Hz, 6H, phen), 8.34 (s, 6H, phen), 7.78 (dd, J = 6.0 Hz, J = 8.2, Hz, 6H, phen), 7.52 (d, 1H, J = 5.6 Hz, 6H, phen).

Elemental analysis data for C_36_H_24_N_6_SO_4_Co × 7H_2_O (%): calculated C 52.2, H 4.66, N 10.23; found C 51.72, H 4.60, N 10.29. 

The VIS spectra of Co(phen)_3_^2+^ and Co(phen)_3_^3+^ are exhibited in Figure 1b.

### 4.4. Synthesis of (5-Acrylamido-1,10-phenanthroline)Bis(2,2′-bipyridine)Cobalt(II) Sulfate [Co(Acphen)(Bpy)_2_^2+^]

5-аcrylamide-1,10-phenanthroline was obtained as described earlier [13]. In this synthesis, 1,10-phenanthroline-5-amine (58.6 mg = 0.300 mmol) was mixed with a solution of tetramethylethylenediamine (29.9 μL, 23.2 mg = 0.200 mmol) in 9 mL of tetrahydrofuran (dried from water). The suspension was thoroughly mixed for 1 h at 10 °C, after which solution of acryloyl chloride (28.5 μL, 31.9 mg = 0.352 mmol) in 1 mL of tetrahydrofuran was added to it dropwise and the mixture was kept stirring for 20 h at 10 °C. The mixture was evaporated, and the target substance was isolated from the residue by column chromatography (silica gel, ethanol/dichlormethane 1:7), which resulted in obtaining 5-аcrylamide-1,10-phenanthroline (37.0 mg = 0.148 mmol) at a 49% yield. Parameters of the ^1^H NMR spectra: δ 10.33 (s, 1H, NH), 9.13 (d, J = 2.8 Hz, 1H, phen-H), 9.03 (d, J = 4 Hz, 1H, phen-H), 8.60 (dd, J = 8 Hz, J = 0,8 Hz, 1H, phen-H), 8.45 (dd, J = 8 Hz, J =1,2 Hz, 1H, phen-H), 8.29 (s, 1H, phen-H), 7.87–7.78 (m, 1H, phen-H), 7.77–7.68 (m, 1H, phen-H), 6.79–6.64 (m, 1H, CH=CH_2_), 6.35 (d, J = 16.8 Hz, 1H, CH=CH_2_), 5.86 (d, J = 10.4 Hz, 1H, CH=CH_2_). Elemental analysis data for C_15_H_11_N_3_O (%): calculated C 72.28, H 4.45, N 16.86; found C 72.31, H 4.50, N 16.83. The parameters of the ^1^H NMR spectra were similar to the reported parameters [13].

To synthesize [Co(acphen)(bpy)_2_^2+^] (Scheme 2), 5-acrylamide-1,10-phenanthroline (123.7 mg = 0.5 mmol) and 2,2′-bipyridine (156.2 mg = 1 mmol) were mixed in 10 mL of methanol. The resulting mixture was mixed with 500 μL of 1 M solution of cobalt(II) sulfate (77.50 mg = 0.5 mmol). The obtained mixture was kept stirring for 3 h at 50 °C and evaporated in vacuum. The target substance was isolated from the residue by column chromatography (Sephadex LH-20, benzene/ethanol (1:1)). The eluate was evaporated to give Co(acphen)(bpy)_2_^2+^, (260 mg = 0.370 mmol) as a light brown powder with the 65% yield.

Parameters of the ^1^H NMR spectra: δ 9.13 (d, J = 2.8 Hz, 1H, phen-H), 8.90 (d, J = 4 Hz, 1H, phen-H), 8.68 (d, J = 7.6 Hz, 4H, bpy-H), 8.60 (dd, J = 8 Hz, J = 0,8 Hz, 1H, phen-H), 8.45 (dd, J = 8 Hz, J =1,2 Hz, 1H, phen-H), 8.40 (t, J = 8.4 Hz, 4H, bpy-H), 8.29 (s, 1H, phen-H), 7.87–7.78 (m, 1H, phen-H), 7.77–7.68 (m, 1H, phen-H), 7.63 (t, J = 6.4 Hz, 4H, bpy-H), 7.31 (d, J = 5.6 Hz, 4H, bpy-H), 6.6–6.45 (m, 1H, CH=CH2), 6.39 (d, J = 16.8 Hz, 1H, CH=CH2), 5.93 (d, J = 10.4 Hz, 1H, CH=CH_2_).

The VIS spectra of Co(acphen)(bpy)_2_^2+^ and Co(acphen)(bpy)_2_^3+^ are exhibited in Figure 1c.

### 4.5. Synthesis of (5-Acrylamido-1,10-phenanthroline)Bis(1,10-phenanthroline)Cobalt(II) Sulfate [Co(Acphen)(Phen)_2_^2+^]

To synthesize [Co(acphen)(phen)_2_^2+^] (see Scheme 2), 5-acrylamide-1,10-phenanthroline (123.7 mg = 0.5 mmol) and 1,10-phenanthroline (182.2 mg = 1 mmol) were mixed in 10 mL of methanol. The resulting mixture was mixed with 500 μL of 1 M solution of cobalt(II) sulfate (77.50 mg = 0.5 mmol). The obtained mixture was kept stirring for 3 h at 50 °C and evaporated in vacuum. The target substance was isolated from the residue by column chromatography (Sephadex LH-20, benzene/ethanol (1:1)). The eluate was evaporated to give Co(acphen)(phen)_2_^2+^ (254 mg = 0.365 mmol) as a brown powder with a 71% yield.

Parameters of the ^1^H NMR spectra: 9.00–8.85 (m, 6H, phen-H), 8.11–8.00 (m, 5H, phen-H), 7.84–7.78 (m, 6H, phen-H), 7.35–7.20 (m, 6H phen-H), 6.60 (t, 1H, J = 8.14 Hz, CH=CH_2_), 5.81 (m, 2H, CH=CH_2_).

Elemental analysis data for C_39_H_27_N_7_SO_5_Co × 5H_2_O (%): calculated C 54.80, H 4.36, N 11.47; found C 54.75, H 4.12, N 11.62. 

The VIS spectra of Co(acphen)(phen)_2_^2+^ and Co(acphen)(phen)_2_^3+^ are exhibited in Figure 1d.

### 4.6. Synthesis of Tris(5-acrylamido-1,10-phenanthroline) Cobalt(II) Sulfate [Co(Acphen)_3_^2+^]

For this synthesis, 5-acrylamido-1,10-phenanthroline (249.3 mg = 1 mmol) was mixed with 10 mL of methanol. To the resulting mixture was added with stirring 333 μL of 1 M solution of cobalt(II) sulfate (51.67 mg = 0.333 mmol). The obtained mixture was kept stirring for 3 h at 50 °C and evaporated in vacuum. The obtained brown residue was purified using column chromatography (Sephadex LH-20, benzene/ethanol (1:1)). The eluate was evaporated to give Co(acphen)_3_^2+^ as a brown powder with a 63% yield. 

The parameters of the ^1^H NMR spectra: 8.98–8.92 (m, 3H, phen-H), 8.83–8.73 (m, 3H, phen-H), 8.23–8.16 (m, 3H, phen-H), 8.10–8.03 (m, 3H phen-H), 7.84–7.74 (m, 3H phen-H), 7.36–7.32 (m, 3H phen-H), 7.25–7.13 (m, 3H phen-H), 6.61–6.54 (m, 3H, CH=CH_2_), 6.38 (d, 3H, J = 16.8 Hz, CH=CH_2_), 5.94 (d, 2H, J = 10.4 Hz, CH=CH_2_).

Elemental analysis data for C_45_H_33_CoN_9_O_7_S × 9H_2_O(%): calculated C 50.75, H 4.83, N 11.84; found C 50.89, H 4.56, N 12.94. 

The VIS spectra of Co(acphen)_3_^2+^ and Co(acphen)_3_^3+^ are exhibited in Figure 1e.

### 4.7. Synthesis of the Gel Cationite

To synthesize this gel, we mixed together 200 μL of 5 M acrylamide (71.08 mg = 1 mmol), 100 μL of 1 M 2-acrylamido-2-methyl-1-propanesulfonic acid sodium salt (23.02 mg = 0.1 mmol), and 100 μL of 200 mM *N*,*N*′-methylenbisacrylamide (3.08 mg = 0.02 mmol). The volume of the mixture was increased to 480 μL by adding water, and then the mixture was thoroughly mixed and degassed under vacuum. To start polymerization, 10 μL of 1 M ammonium persulfate (2.28 mg = 0.01 mmol) and 10 μL of 1 M tetramethylethylenediamine (1.16 mg = 0.01 mmol) were added. Polymerization continued for 1 h at room temperature, after which the resulting gel was incubated 3 times for 1 day in 10 mL of distilled water to remove low molecular compounds.

### 4.8. Synthesis of the Gels 1 and 2 (Immobilization of Co(Bpy)_3_^2+^ or Co(Phen)_3_^2+^ in the Gel Cationite)

To immobilize the catalyst, 1 mL of the gel was kept in 10 mL of 10 mM solution of the corresponding complex for 24 h. The resulting gel was incubated for 1 day in 10 mL of distilled water to remove the non-immobilized catalyst.

### 4.9. Synthesis of Gel 3

To synthesize gel 3, we mixed together 250 μL of 5 M acrylamide (88.85 mg = 1.25 mmol), 62.6 μL of 200 mM *N*,*N*′-methylenbisacrylamide (1.93 mg = 0.0125 mmol), and 35.83 mg of (5-acrylamido-1,10-phenanthroline)bis(2,2′-bipyridine) cobalt(II) sulfate (0.05 mmol). The volume of the mixture was increased to 480 μL by adding water, and then the mixture was thoroughly mixed and degassed under vacuum. To start polymerization, 10 μL of 1 M ammonium persulfate (2.28 mg = 0.01 mmol) and 10 μL of 1 M tetramethylethylenediamine (1.16 mg = 0.01 mmol) were added. Polymerization continued for 1 h at room temperature, after which the resulting gel was incubated 3 times for 1 day in 10 mL of distilled water to remove low molecular compounds.

### 4.10. Synthesis of Gel 4

To synthesize gel 4, we mixed together 200 μL of 5 M acrylamide (71.08 mg = 1 mmol), 50 μL of 200 mM *N*,*N*′-methylenbisacrylamide (1.54 mg = 0.01 mmol) and 30.74 mg of (5-acrylamido-1,10-phenanthroline)bis(1,10-phenanthroline) cobalt(II) sulfate (0.04 mmol). The volume of the mixture was increased to 480 μL by adding water, and then the mixture was thoroughly mixed and degassed under vacuum. To start polymerization, 5 μL of 1 M ammonium persulfate (1.14 mg = 0.005 mmol) and 5 μL of 1 M tetramethylethylenediamine (0.58 mg = 0.005 mmol) were added. Polymerization continued for 1 h at room temperature, after which the resulting gel was incubated 3 times for 1 day in 10 mL of distilled water to remove low molecular compounds.

### 4.11. Synthesis of Gel 5

To synthesize gel 5, we mixed together 250 μL of 5 M acrylamide (88.85 mg = 1.25 mmol) and 22.57 mg of tris(5-acrylamido-1,10-phenanthroline) cobalt(II) sulfate (0.025 mmol). The volume of the mixture was increased to 475 μL by adding water, and then the mixture was thoroughly mixed and degassed under vacuum. To start polymerization, 12.5 μL of 1 M ammonium persulfate (2.85 mg = 0.0125 mmol) and 12.5 μL of 1 M tetramethylethylenediamine (1.45 mg = 0.0125 mmol) were added. Polymerization continued for 1 h at room temperature, after which the resulting gel was incubated 3 times for 1 day in 10 mL of distilled water to remove low molecular compounds.

### 4.12. Experimental Setup

Oscillations of the pieces of the gels in CFBZ solution were observed using a microscope equipped with a color camera connected to a personal computer. A Petri dish with the pieces of the gels was illuminated from below with a LED light source. Recording of light transmission through the pieces of the gels and geometrical measurements of the pieces of the gels were performed using QCapture ProS2 software. For each piece of gel, the space–time plot was constructed for cross-sections indicated by arrows (Figure 3). The sequence of the cross-sections (recorded every second) in time was combined together to create a space–time plot for analysis of the linear dimensions and the periods of the oscillations.

### 4.13. Data Processing

To enhance visualization, the mean intensity of pixels within the gel region was plotted, as color changes were not always pronounced. An algorithm based on Python was devised, primarily leveraging functions from the ‘OpenCV’ library, to transform video recordings into the aforementioned graphs. For user convenience, the Python code was executed using Jupyter notebooks (open-source software, v7.2), facilitating interactive data analysis. The algorithm encompasses two primary stages, applied to each frame of the recording. During the initial stage, computer vision methodologies are employed to isolate the gel spot from the background. Segmentation was effectively accomplished in the HSV color space by establishing specific thresholds for hue and saturation channels, derived from the color histogram of these channels. Subsequently, in the second stage, the average pixel intensity within the segmented area was computed using the value channel, which denotes pixel brightness. The average pixel intensity, denoted by intensity unit (IU) on the graphs, spans from 0 to 255.

## Data Availability

The raw/processed data required to reproduce these findings cannot be shared at this time as the data also form part of an ongoing study.

## Supplementary Materials

The following supporting information can be downloaded at: https://www.mdpi.com/article/10.3390/gels10090552/s1, Movies S1 and S2.

## References

[1] Yoshida R. (2022). Creation of softmaterials based on self-oscillating polymer gels. Polym. J..

[2] Kim Y.S., Tamate R., Akimoto A.M., Yoshida R. (2017). Recent developments in self-oscillating polymeric systems as smart materials: From polymers to bulk hydrogels. Mater. Horiz..

[3] Mallphanov I.L., Vanag V.K. (2021). Chemical micro-oscillators based on the Belousov–Zhabotinsky reaction. Russ. Chem. Rev..

[4] Aishan Y., Yalikun Y., Shen Y., Yuan Y., Amaya S., Okutaki T., Osaki A., Maeda S., Tanaka Y. (2021). A chemical micropump actuated by self-oscillating polymer gel. Sens. Actuators B Chem..

[5] Murase Y., Hidaka M., Yoshida R. (2010). Self-driven gel conveyer: Autonomous transportation by peristaltic motion of self-oscillating. Sens. Actuators B Chem..

[6] Adamatzky A., Costello B.D.L., Asai T. (2005). Reaction-Diffusion Computers.

[7] Steinbock O., Kettunen P., Showalter K. (1996). Chemical Wave Logic Gates. Phys. Chem..

[8] Tateyama S., Shibuta Y., Yoshida R. (2008). Direction Control of Chemical Wave Propagation in Self-Oscillating Gel Array. Phys. Chem. B.

[9] Mallphanov I.L., Vanag V.K., Lavrova A.I. (2024). A new visible light-sensitive, oxygen-tolerant photoinitiator system for the synthesis of polyacrylamide gels by maskless photopolymerization. Mendeleev Commun..

[10] Yuan P., Kuksenok O., Gross D.E., Balazs A.C., Moore J.S., Nuzzo R.G. (2013). UV patternable thin film chemistry for shape and functionally versatile self-oscillating gels. Soft Matter.

[11] Yoshida R., Takahashi T., Yamaguchi T., Ichijo H. (1996). Self-Oscillating Gel. J. Am. Chem. Soc..

[12] Ueki T., Yoshida R. (2014). Recent aspects of self-oscillating polymeric materials: Designing self-oscillating polymers coupled with supramolecular chemistry and ionic liquid science. Phys. Chem. Chem. Phys..

[13] Mallphanov I.L., Vanag V.K. (2022). Self-oscillating gels based on novel catalyst for the Belousov–Zhabotinsky reaction. Mendeleev Commun..

[14] Belousov B.P. (1959). Collection of Short Papers on Radiation Medicine.

[15] Field R.J., Koros E., Noyes R.M. (1972). Oscillations in Chemical Systems. 2. Thorough Analysis of Temporal Oscillation in Bromate-Cerium-Malonic Acid System. J. Am. Chem. Soc..

[16] Toth R., Taylor A.F. (2006). The tris(2,2′-bipyridyl)ruthenium-catalysed Belousov–Zhabotinsky reaction. Prog. React. Kinet. Mech..

[17] Yoshida R., Ueki T. (2014). Evolution of self-oscillating polymer gels as autonomous polymer systems. NPG Asia Mater..

[18] Arimura T., Mukaia M. (2014). A self-oscillating gel actuator driven by ferroin. Chem. Commun..

[19] Ren J., Tao L., He J.H., Zhang A.X., Yang W. (2018). Synchronous volume and color self-oscillating gels based on chemomechanical coupling. J. Polym. Res..

[20] Konotop I.Y., Nasimova I.R., Rambidi N.G., Khokhlov A.R. (2009). Self-oscillatory systems based on polymer gels. Polym. Sci. Ser. B.

[21] Lagunova O.V., Vanag V.K., Mallphanov I.L. (2023). Fe(bathophen)_2_(phen)-based self-oscillating gel driven by the Belousov–Zhabotinsky reaction. Mendeleev Commun..

[22] Safonov D.A., Mallphanov I.L., Sychev A.V., Postnikov E.B., Lavrova A.I. (2024). On phase correspondence between chemical oscillations in liquid Belousov-Zhabotinsky mixture filling catalyst-containing matrix and resulted gel’s chemomechanical oscillations. J. Mol. Liq..

[23] Handlířová M., Tockstein A. (1980). New catalysts for Belousov-Zhabotinskii reaction. Collect. Czechoslov. Chem. Commun..

[24] Szalada D.J., Creutz C., Mahajan D., Sutin N. (1983). Electron-transfer barriers and metal-ligand bonding as a function of metal oxidation state. 2. Crystal and molecular structures of tris(2,2′-bipyridine)cobalt(II) dichloride-2-water-ethanol and tris(2,2′-bipyridine)cobalt(I) chloride-water. Inorg. Chem..

[25] Tao B., Xia H., Zhu Y.-F., Wang X.J. (2012). Synthesis, crystal structure and electrochemical properties of [Co(phen)_3_] · (H_3_btec) · (H_2_btec)_0_._5_ · DMF · 6H_2_O. Inorg. Chem..

[26] Nia Y.N., Farahani P., Sabzyan H., Zendehdel M., Oftadeh M. (2014). A combined computational and experimental study of the [Co(bpy)_3_]^2+/3+^ complexes as one-electron outer-sphere redox couples in dye-sensitized solar cell electrolyte media. Phys. Chem. Chem. Phys..

[27] Showalter K., Noyes R.M., Bar-Eli K.A. (1978). Modified Oregonator Model Exhibiting Complicated Limit Cycle Behavior in a Flow System. J. Chem. Phys..

[28] Saczko-Brack D., Warchol E., Rogez B., Kröss M., Heissler S.M., Sellers J.R., Batters C., Veigel C. (2016). Self-organization of actin networks by a monomeric myosin. Proc. Natl. Acad. Sci. USA.

[29] Zhang Y., Zhou N., Akella S., Kuang Y., Kim D., Schwartz A., Bezpalko M., Foxman B.M., Fraden S., Epstein I.R. (2013). Active Cross-Linkers that Lead to Active Gels. Angew. Chem Int. Ed..

[30] Aizenberg M., Okeyoshi K., Aizenberg J. (2018). Inverting the Swelling Trends in Modular Self-Oscillating Gels Crosslinked by Redox-Active Metal Bipyridine Complexes. Adv. Funct. Mater..

[31] Konotop I.Y., Nasimova I.R., Rambidi N.G., Khokhlov A.R. (2011). Chemomechanical oscillations in polymer gels: Effect of the size of samples. Polym. Sci. Ser. B.

